# What Matters 2 Adults (WM2Adults): Understanding the Foundations of Aboriginal and Torres Strait Islander Wellbeing

**DOI:** 10.3390/ijerph18126193

**Published:** 2021-06-08

**Authors:** Gail Garvey, Kate Anderson, Alana Gall, Tamara L. Butler, Joan Cunningham, Lisa J. Whop, Michelle Dickson, Julie Ratcliffe, Alan Cass, Allison Tong, Brian Arley, Kirsten Howard

**Affiliations:** 1 Menzies School of Health Research, Charles Darwin University, Darwin 0810, Australia; Gail.Garvey@menzies.edu.au (G.G.); alana.gall@menzies.edu.au (A.G.); tamara.butler@menzies.edu.au (T.L.B.); Joan.Cunningham@menzies.edu.au (J.C.); lisa.whop@anu.edu.au (L.J.W.); alan.cass@menzies.edu.au (A.C.); brian.arley@menzies.edu.au (B.A.); kirsten.howard@sydney.edu.au (K.H.); 2 School of Public Health, University of Queensland, Brisbane 4000, Australia; 3 Sydney School of Public Health, The University of Sydney, Sydney 2006, Australia; michelle.dickson@sydney.edu.au (M.D.); allison.tong@sydney.edu.au (A.T.); 4 National Centre for Epidemiology and Population Health, Australian National University, Canberra 2601, Australia; 5 Health and Social Care Economics Group, Caring Futures Institute, Flinders University, Adelaide 5042, Australia; julie.ratcliffe@flinders.edu.au; 6 Menzies Centre for Health Policy and Economics, The University of Sydney, Sydney 2006, Australia

**Keywords:** Aboriginal and Torres Strait Islander people, Australian, Indigenous, wellbeing, quality of life

## Abstract

Aboriginal and Torres Strait Islander people experience a greater range of health and social disadvantages compared to other Australians. Wellbeing is a culturally-bound construct, and to date, a national evidence base around the components of wellbeing for Aboriginal and Torres Strait Islander people is lacking. Understanding and measurement of wellbeing for this population is critical in achieving health equity. This paper aims to identify and describe the foundations of wellbeing for Aboriginal and Torres Strait Islander adults. This national qualitative study was underpinned by an Indigenist research approach which privileges the voices of Aboriginal and Torres Strait Islander people. Aboriginal and Torres Strait Islander adults were purposively recruited from around Australia between September 2017 and September 2018 to participate in Yarning Circles, led by Aboriginal and Torres Strait Islander researchers. Yarning Circles were audio recorded, transcribed and analyzed. A Collaborative Yarning Methodology was used, which incorporated reflexive thematic analysis to identify and describe the foundations of wellbeing reported by participants. A total of 359 Aboriginal and Torres Strait Islander adults participated. Our analysis revealed five foundations of wellbeing: belonging and connection; holistic health; purpose and control; dignity and respect; and basic needs. These foundations were deeply interwoven by three interconnected aspects of Aboriginal and Torres Strait Islander life: family, community and culture. The findings of this study will substantially aid our efforts to develop a new wellbeing measure for Aboriginal and Torres Strait Islander adults. The iterative Indigenist methods used in this study provide a robust research methodology for conducting large-scale, nationally-relevant qualitative research with Aboriginal and Torres Strait Islander people. Policies and practices that are informed by our results have the potential to address outcomes that are meaningful for Aboriginal and Torres Strait Islander people.

## 1. Introduction

Variously referred to as quality of life (QoL), subjective well-being, and social and emotional wellbeing, wellbeing [1] is a broad and multidimensional concept defined as ‘*an individual’s perception of their position in life in the context of the culture and value systems in which they live, and in relation to their goals, expectations, standards and concerns*’ [2], p. 1403. There is increasing interest by clinicians, researchers and policy makers in measuring wellbeing as a standard health outcome measure [3,4,5].

Understandings of wellbeing are culturally bound, which means that measures of wellbeing need to account for differences in the conception and experience of wellbeing among different cultural groups. This said, the preponderance of existing wellbeing measures have been developed within Western individualistic paradigms [3]. While there has been increasing interest in developing culturally-specific wellbeing measures, most often, the wording in existing instruments is simply translated for culturally and linguistically diverse respondents [6], with the implicit assumption that culture and context have no effect on the relevance of the content of wellbeing instruments [7]. However, this approach fails to capture critical concepts and dimensions of wellbeing relevant to specific populations [8,9].

There is an imperative to understand wellbeing from an Aboriginal and Torres Strait Islander cultural perspective and to have measures available that assess holistic indicators of wellbeing and prioritise the perspectives and values of Aboriginal and Torres Strait Islander people [10]. This is fundamental in addressing the health and wellbeing inequalities experienced by Aboriginal and Torres Strait Islander people, which have persisted, and in some cases increased, for many decades [11]. Aboriginal and Torres Strait Islander people account for 3.3% of the total Australian population and most live in major cities or regional areas; however, they are significantly overrepresented in the population who live in remote and very remote areas (30%). Aboriginal and Torres Strait Islander people have poorer health outcomes and lower life expectancy, which is approximately ten years less than for other Australians [12,13]. These inequalities persist due to the ongoing legacy of colonisation, ongoing marginalisation, social inequality, racism and the social and political circumstances facing Aboriginal and Torres Strait Islander people [14].

Eliminating these disparities has become a priority for national health policies and government initiatives, such as Closing the Gap; however, progress has been slow and many targets have not been met [15,16]. The Australian Government Closing the Gap Report states, ‘*There is more work to be done to close the gap in health inequality, including by harnessing the strength of culture as an underlying determinant of good health through identity and belonging, supportive relationships, resilience and wellbeing*’ [11], p. 8. This work must include measures of wellbeing that are holistic, valid and robust, and include concepts and dimensions that are relevant to Aboriginal and Torres Strait Islander people [17]. While this may appear self-evident, there is a notable absence of measures of wellbeing to address these targets [18], and none that are embedded in the aspects of life that are important to Aboriginal and Torres Strait Islander people. A recent global systematic review reported a lack of Indigenous specific quality of life measures and highlighted the need for such measures [3].

There is a growing interest, both in Australia and globally, in developing measures to quantify Indigenous peoples’ wellbeing that acknowledge and value their cultures and ways of knowing, being and doing [10]. A handful of Australian studies, largely conducted within discrete Aboriginal and Torres Strait Islander communities, settings or populations, have identified some important aspects of wellbeing [19,20,21,22]. However, to date, no studies have comprehensively identified the foundations of wellbeing relevant to, and valued by, Aboriginal and Torres Strait Islander adults at a national level. Understanding the parts of life that comprise and support the wellbeing of Aboriginal and Torres Strait Islander adults is a critical first step in developing measures of wellbeing that are culturally relevant to this population. Given the enduring process of colonial disempowerment and marginalisation of Aboriginal and Torres Strait Islander people, the use of Indigenist methodologies and a strengths-based approach may avoid further subjugation of this group [10,23].

This paper aims to address the critical evidence gap by identifying and describing the foundations of wellbeing for Aboriginal and Torres Strait Islander adults. This paper presents the findings of a large, nationally-relevant qualitative study, which employed a strengths-based, Indigenist research approach to understand what comprises and supports the wellbeing of Aboriginal and Torres Strait Islander adults.

## 2. Materials and Methods

### 2.1. An Indigenist Methodology

This qualitative study is the first of three phases of the ‘What Matters 2 Adults’ (WM2Adults) Research Project. The WM2Adults Project aims to develop a new national instrument to measure wellbeing, in order to more effectively inform decision making to improve the health and wellbeing of Aboriginal and Torres Strait Islander people. The overall methods of the WM2Adults Program are described elsewhere [24]. In brief, the first phase (reported here) used qualitative research methods underpinned by an Indigenist research methodology which is informed by three core principles: (1) notions of resistance as a part of Aboriginal and Torres Strait Islander peoples’ struggle for self-determination; (2) leadership of Aboriginal and Torres Strait Islander researchers in representing their communities to achieve self-determination; and (3) ensuring political integrity and privileging the voices of Aboriginal and Torres Strait Islander people [25]. The methods for this study are reported in detail below; informed by the *Consolidated criteria for strengthening reporting of health research involving Indigenous peoples* (the CONSIDER statement) [26]. The use of a strengths-based, Indigenist methodology in this study is critical in ensuring that Aboriginal and Torres Strait Islander people and values are prioritised in the conduct and the outcomes of the research. This methodology avoids the further subjugation of Aboriginal and Torres Strait Islander people, and ensures autonomy, inclusion and benefit to Aboriginal and Torres Strait Islander people across all aspects of the research.

### 2.2. Research Team

Our team acknowledges the importance of reflexively considering and describing our backgrounds, perspectives and values that we each bring to the project [27,28]. The research team includes Aboriginal and Torres Strait Islander researchers (GG, AG, TB, MD, LJW, BA), qualitative researchers (GG, KA, AG, TB, MD, AT), health economists (KH, JR), and epidemiologists (AC, JC, LJW) working in Aboriginal and Torres Strait Islander health research.

### 2.3. Indigenous Governance

An Indigenous Project Advisory Group (IPAG) was established at the WM2Adults Program inception and consisted of representatives of key Aboriginal and Torres Strait Islander stakeholder groups and community members. The IPAG guided the overall program of work including our research methods and data interpretation. An Indigenous Researchers Group (IRG) was also formed and comprised of Aboriginal and Torres Strait Islander study investigators and research staff, who provided additional in-depth guidance with the data analysis and interpretation.

### 2.4. Participants and Data Collection

Aboriginal and Torres Strait Islander adults (18 years of age or older at the time of recruitment) were purposively recruited between September 2017 and September 2018. Participants were recruited from a wide range of geographic locations including major cities, regional and remote areas from seven states and territories across Australia to ensure we captured a diverse range of views on aspects of wellbeing that are important to Aboriginal and Torres Strait Islander people. To maximize diversity, we identified organisations and groups from across Australia, to seek their interest in supporting participant recruitment for the study. The organisations approached varied greatly, with examples including community health services, sporting and recreational groups, men’s and women’s groups, Elders’ groups, community art and cultural centres and other organisations. Engagement with sites prior to the data collection visit was usually conducted by one researcher (BA) by phone and email.

At each participating site, a local contact assisted with recruiting participants and organising with the research team a suitable venue, time and day to conduct the field work, which largely consisted of Yarning Circles, and securing the services of an interpreter if that was deemed necessary by the contact. An interpreter was only required at one remote site and they assisted with communication during the Yarning Circle. ‘Yarning’ is a recognised culturally-appropriate style of communication and is used to build trust, facilitate connection between researchers and participants, and gather information through sharing knowledge [29,30,31,32]. This method of gathering information respects Aboriginal and Torres Strait Islander peoples’ oral traditions and values, and privileges Aboriginal and Torres Strait Islander knowledge [29,30,31]. Face to face Yarning Circles were held with groups of ~8–10 Aboriginal and Torres Strait Islander adults and ranged in duration from 60–120 min. Individual Yarns were held with a small number of participants who preferred to speak one-on-one. The Yarning Circles and Individual Yarns (hereafter called Yarns) were all conducted by an Aboriginal and Torres Strait Islander researcher trained in qualitative research and assisted by an Aboriginal and Torres Strait Islander or non-Indigenous researcher. Yarns were conducted until data saturation was reached. All participants were asked whether they wished to receive a transcript or summary of their Yarn; those who did were sent a copy for their review. All participants received updates throughout the study. Before commencing the formal Yarn, the Aboriginal and Torres Strait Islander researcher engaged in a Social Yarn with participants to introduce themselves; their language group and connection with other Aboriginal and Torres Strait Islander people and communities [33]. This Social Yarn was important in breaking the ice, building trust and helping both the participants and the researchers to feel comfortable [33]. As an important aspect of cultural protocol, food was shared during the Social Yarn. Following this, the researcher described the purpose of the study, provided an information sheet and consent form, and answered any questions regarding the study. Informed consent was obtained, including consent to audio record the discussions. Sociodemographic information, such as age, gender, place of residence (and postcode), marital status, level of education and health conditions, was collected using participant self-report. All participants were reimbursed for their time with a $50 gift voucher. The participants’ postcode and/or name of place of residence was used to assign participants to a remoteness level, based on the 2006 Australian Remoteness Index of Areas (ARIA), which is a measure of the accessibility of goods, services and opportunities for social interactions, based on road distance to the nearest service centre [34]. Geographical areas are classified as ‘Major City’ (relatively unrestricted accessibility to goods, services and social opportunities), ‘Inner Regional’ (some restrictions to accessibility), ‘Outer Regional’ (significant restrictions to accessibility), ‘Remote’ (very restricted accessibility), or ‘Very Remote’ (very little accessibility).

The Yarning question guide was informed by our systematic reviews [3,9] and previous studies [17,35] and included open ended questions developed and refined by the research team and the IPAG. The guide included questions to encourage participants to identify and describe the parts of life that they consider to be important to their wellbeing and what it means to live a ‘good life’ such as: *What matters in your life? What’s important to you in your life? What are some things that affect your quality of life? Are you able to identify aspects of your life that are important to your quality of life at the moment and could you rank them? Is there a word or words that best describe what we’ve been talking about?* Participants were then guided to reflect on, discuss and describe the impact of those parts of life on their wellbeing. These questions were followed by a list of prompts on some aspects of wellbeing that had been identified as important in a comprehensive literature review conducted by our research team [9].

### 2.5. Ethics

Formal ethics approvals were obtained from the Human Research Ethics Committee of the Northern Territory Department of Health and Menzies School of Health Research (Ref: 2017-2855 and Ref. 2019-3333); University of Sydney Human Research Ethics Committee (Ref: 2017/724 and Ref. 2019/672); Central Australian Aboriginal Congress Aboriginal Corporation; Central Australian Human Research Ethics Committee; Western Australian Aboriginal Health Ethics Committee (Ref: 833); Aboriginal Health & Medical Research Council (Ref: 1340/17); Aboriginal Health Council of South Australia’s Aboriginal Health Research Ethics Committee (Ref: 04-17-741); St Vincent’s Hospital Melbourne Human Research Ethics Committee (Ref: 034/18); UTS Human Research Ethics Committee (Ref: ETH194460); and the Charles Darwin University Human Research Ethics Committee (Ref: H19059).

### 2.6. Data Analyses

In line with an Indigenist research approach, the voices of Aboriginal and Torres Strait Islander people were privileged in all aspects of the analysis [26]. There were a number of collaborative and iterative steps in our analysis process, which used a Collaborative Yarning Methodology (CYM) [36] led by Aboriginal and Torres Strait Islander researchers.

The audio recordings from the Yarns were transcribed verbatim and imported into NVIVO12 software [37] for analysis. An adapted grounded theory approach and reflexive thematic analysis [38] was used, whereby two researchers (KA, TB) initially reviewed a sample of the transcripts line-by-line to inductively generate a draft thematic framework relating to the parts of life identified as important to the wellbeing of participants. The IRG met and used a CYM approach to iteratively review and revise the draft thematic framework via reflection on extracted raw data and discussion. Two researchers (KA, AG) then re-coded the transcripts in line with the revised thematic framework, and through a process of constant comparisons, analytical themes and coding were cross-checked with a third senior researcher (GG) to develop a final unified coding approach. When the coding was complete, three researchers wrote summaries of the themes (KA, AG, GG). The IRG then held three meetings over a six-week period to Yarn and review the themes and distil the key points for each theme. The IPAG met and members provided feedback and suggested revisions on the phrasing of the key points.

In order to explore whether themes were specific to particular geographic areas, the number of times that themes were coded across different states/territories and across urban, regional and remote sites was examined. While it was not possible to stratify the results by language groups, we also examined, using a similar approach to geographic area, whether different themes emerged for Aboriginal participants compared with Torres Strait Islander participants.

## 3. Results

### 3.1. Profile of Participants

A total of 359 Aboriginal and Torres Strait Islander adults participated in the Yarning Circles (*n* = 353) and Individual Yarns (*n* = 6). The characteristics of the participants are described in Table 1. In summary, 57.1% of participants in our study were female and the age of participants ranged from 18 to 92 years, with a median age of 50 years. Most participants were Aboriginal (84.4%), resided in regional areas (57.1%), had an educational attainment level equivalent to grade 10 or below (53.9%), and spoke English as their main language at home (80.5%).

### 3.2. Qualitative Results

Our analysis of the qualitative data revealed five foundations of wellbeing for Aboriginal and Torres Strait Islander adults: *belonging and connection; holistic health; purpose and control; dignity and respect; and basic needs*. Each of these five foundations were interwoven with three interconnected aspects of Aboriginal and Torres Strait Islander life: *family, community and culture*. These five emergent themes are described below in the context of their relationships to family, community and culture. There is substantial interconnection and overlap across the five themes, which signifies the deeply interconnected nature of being and wellbeing for Aboriginal and Torres Strait Islander people. This characteristic of connectedness across the different parts of life and parts of wellbeing strongly aligns with the traditional Aboriginal and Torres Strait Islander conception of health, which focuses on the whole rather than the individual parts in isolation. Moreover, the strong connected nature of wellbeing found here reflects the collectivist values of Aboriginal and Torres Strait Islander cultures, which emphasize the interests of the group more than those of the individual. We also found that the most commonly mentioned themes were highly consistent across geographic areas, and between Aboriginal and Torres Strait Islander participants; thus, we proceeded with the analysis as a single group of themes.

Participant quotes are included throughout the results to illustrate and exemplify the findings. Due to the deeply connected nature of the components of wellbeing found here, it should be noted that many of the quotes have relevance to multiple themes. While individual participants are not identified within Yarning Circles, each of the included quotes is labelled with basic details about the Yarn site—the state/territory in which the Yarn was held and the level of remoteness of the site [39].

#### 3.2.1. Belonging and Connection

Belonging and connection was the broadest theme and the theme most commonly spoken about by participants. While belonging and connection was predominantly associated with an individual’s relationships and bonds with family and community, the importance of culture in developing and maintaining a sense of shared experience and understanding was also highlighted.

Family or ‘mob’ were commonly spoken about in broad and inclusive terms, encompassing extended family, community and others. Family was described as supporting wellbeing through a shared sense of reciprocal belonging and connection. An individual’s wellbeing was often described as being directly affected by the wellbeing of their family.

*‘They say, you are my mob and that. You could be second or third or fourth cousin, they’re still your mob or I’m one of your mob and just as great as Aboriginal culture. Extended family. Blood and friends.’*

(Participant, New South Wales, Inner Regional)

It was explained that people just feel comfortable when they are around their own mob. Family was described by many participants as their main source of love and care, with family members tending to look after one another and offer company, comfort and understanding. Family also offers people important opportunities for healing, laughter and an emotional refuge from the challenges people face in life.

*‘We laugh, we joke, we sing with one another as a group and that will make us happy, will make us being together as one and yeah.’*

(Participant, New South Wales, Inner Regional)

The participants derived a sense of identity from knowing who their family or mob were. Spending time with family can strengthen identity and cultural connections, while an absence or loss of connection with family was said to also break important ties with culture and Country.

*‘[If] I don’t have family, I don’t have identity. If I don’t have identity, I become unwell.’*

(Participant, Queensland, Major City)

It was said that families are the context in which culture and spirituality are shared, learned, and passed down. Participants described family as the teachers of culture, and it was stressed that connecting with and listening to Elders was centrally important to passing on traditional knowledge. Fostering a strong sense of connection and belonging with one’s family was said to be intrinsically important to Aboriginal and Torres Strait Islander cultural survival. While it was acknowledged that many family connections had been broken due to the forced removals of children and family members through the Stolen Generations, striving to reconnect with family was important to ensure that Aboriginal and Torres Strait Islander cultures continue.

(The *Stolen Generations* are the children of Aboriginal and Torres Strait Islander people who were forcibly removed from their families by the Australian federal and state government agencies and church missions, under acts of their respective parliaments [40]).

Supplementing the role that family provides in supporting belonging and connection, being part of a community was also emphasised as central in facilitating support, sharing and respect. Community connections were said to foster wellbeing through the development and maintenance of friendships, shared experiences, values and identity. These connections bring about the feeling of belonging, which gives a sense of security and comfort.

*‘When you’re around black fellas they’re just like, you’re right [okay]… a sense of belonging.’*

(Participant, New South Wales, Inner Regional)

Participants explained that it feels good to be ‘part of something’, which strongly promotes a sense of wellbeing.

*‘Connection to the community. And I mean, obviously, that’s connection to your culture. So, all those things make for you—make you feel good about yourself. Your self-worth’s better. You feel happy with yourself if you have all those links connected to that one happiness.’*

(Participant, Tasmania, Outer Regional)

In a similar way to the importance of knowing who your family is, it was also emphasised that knowledge about your community and community connections is also important to your identity and sense of self.

*‘Knowing who you’re connected with and knowing what your community is and I think that plays a big bearing on where you stand with your own health and wellbeing, and being connected to being, affiliated to be acknowledged.’*

(Participant, New South Wales, Inner Regional)

Belonging and connection were explained as being inextricably linked to one’s culture. Participating in cultural events and activities was said to be important in bringing people together to have opportunities to connect in positive ways. Activities like hunting, camping, dancing, Corroboree (traditional dance ceremony that may take the form of a sacred ritual or informal gathering), music and art provide shared experiences and opportunities to connect with others, which are considered essential to foster wellbeing.

Participants identified that family is an important conduit for maintaining cultural and spiritual connections. Passing on cultural knowledge to children through families was said to give individuals and families strength and security in knowing who they are and where they come from. Respecting Elders and learning culture through songlines, dances, and traditional stories, was said to give people strength and comfort for the future in knowing that traditional culture is being maintained. These connections with and through culture were described as fostering strong identities and strengthening wellbeing.

*‘But the culture still plays a great part in us older one’s lives. And for me, I was from that—we passed that down to our kids, who then have the onus of spreading that to their kids.’*

(Participant, South Australia, Outer Regional)

Learning and passing on language was said to be critically important to the development of identity, because language is the conduit that connects people to one another, to culture and identifies the relations between people and their place in the world.

*‘That connection to our family is a connection to language, to Country, to know how important that is because it is so important that we have that. So, we never forget where we came from, give us strength for the future.’*

(Participant, Western Australia, Major City)

*‘You remember your mother, your father or your Aunty, your favourite Aunty or Uncle talking to you. And they spoke in a certain way and you clearly remembered everything they said to you, eh. Sometimes I just sit there and marvel and sometimes I’ve cried but it’s always there. It’s hard, I guess. It sounds like that connection to language is quite powerful.’*

(Participant, South Australia, Outer Regional)

*‘I think language really identifies us of who we are and who we belong to in the world… and the coming generation are more aware of how the importance of that plays in our daily lives…’*

(Participant, Queensland, Very Remote)

The importance of maintaining connection with one’s Country was described as paramount to wellbeing. Belonging and connection also manifested in maintaining one’s connection to Country and ancestors. This was described as foundational to Aboriginal and Torres Strait Islander people’s identity.

*‘Connection to Country for me is we can actually get back out and walk the land that our ancestors walked on and do things on the land that our ancestors done and re-educate our younger fellows in the way that our ancestors did.’*

(Participant, Victoria, Major City)

The environmental degradation and man-made destruction of the land and to sacred sites was a source of deep concern and sadness for many participants. It was put forward by participants that protection of the environment is vitally important to wellbeing, due to the deep and reciprocal relationship that Aboriginal and Torres Strait Islander people have with Country. Moreover, maintaining access to cultural sites was described as important to enable people to participate in traditional cultural activities. The loss of this access and degradation of the environment was a source of deep sorrow for many participants.

*‘The country before the white fellows come here, even the white fellows was here, they bought in the fox, they bought in the cats, this bit here had a lots of lizards … and now you can’t find even little ones like that. Now you can’t find one… feral cats… no, no lizard there… no bush tucker…’*

(Participant, South Australia, Outer Regional)

#### 3.2.2. Holistic Health

Being healthy was described by participants as another essential component of wellbeing. In this context, health was described as a holistic and multidimensional state of wellness that was commonly determined and attained via the quality of one’s connections to family, community and culture.

Good health and wellbeing were rooted in strong and positive connections with family. Conversely, broken or disrupted family connections negatively affected the individual’s spiritual wellbeing. Participants reported that disconnection is extremely common in Aboriginal and Torres Strait Islander families and it is often associated with the forcible removal of children from their families under government policies and acts, or when people need to relocate for medical treatment or to access employment and education opportunities.

*‘So when you go home and you’re with family, you’re a lot healthier.’*

(Participant, Northern Territory, Remote)

An individual’s health was also described as being closely connected with the health and wellbeing of family members. This was particularly associated with stress, worry and the pressures of juggling the responsibilities of modern life with the need to care for one’s family.

*‘But I guess it is just that family, when Mum’s okay, I feel okay, my children feel okay. There is no stress in the camp. So, life is good at the moment.’*

(Participant, Northern Territory, Outer Regional)

The importance of having good health was attributed to the need for individuals to be well enough to look after and connect with their family.

*‘If you’re not well in yourself, that means you’re not healthy and you can’t have connection with your family, you can’t connect with your culture.’*

(Participant, Victoria, Major City)

*‘Because if you don’t have health, you can’t look after your family. You can’t look after yourself. Who are you going to help?’*

(Participant, Western Australia, Major City)

*‘If you want to look after your family you have got to look after yourself.’*

(Participant, Western Australia, Major City)

Moreover, being healthy was regarded as important for providing good role models for children, so that good health habits were passed on to the next generation.

*‘I found that when I was younger and I was drinking a lot… I’d send [my children] to the fridge to get me a bottle of beer. Now that’s not teaching. That’s making them learn you can drink. Encourages them. Yeah. I wasn’t teaching them the right thing. I was teaching them the wrong thing. And who’s suffering for it now? My kids.’*

(Participant, New South Wales, Outer Regional)

Balance was described by participants as a critical component in achieving holistic health. Mental health and wellbeing were reported to suffer when an individual put family first, but the care was not reciprocated. Furthermore, achieving a work and family life balance was regarded as important in achieving both individual and family wellbeing.

*‘We should look after ourselves because you can’t pull from an empty cart, but we always give to everyone else I think. So, while our physical health might be good, I think at times our mental health suffers because we are always putting family, community, work above other things.’*

(Participant, New South Wales, Inner Regional)

*‘Yeah, and sometimes it’s important to find a balance between work and not working too much and making sure that you know, everything at home is nice and settled and home life is not being affected because you’re still sitting there at eight o’clock at night working, because that’s not a healthy balance lifestyle.’*

(Participant, Northern Territory, Outer Regional)

Participants described a reciprocal relationship in the role of community in fostering holistic health, whereby the health and wellbeing of the community is impacted on and by the health and wellbeing of the individual. While community groups and connections within the community were said to support an individual’s wellbeing by offering mechanisms to cope with stress, the reverse was also described as true. The state and functioning of the community had a significant bearing on individuals’ wellbeing—both positively and negatively.

*‘That’s why I’ve been sick for nearly 12 months, because of stress and mental health and your health and everything… drugs… yeah. Big problem in town… it’s so out of control. I don’t know what the answer to that is…’*

(Participant, New South Wales, Inner Regional)

Good health and wellbeing were said to be derived through the development of caring, supportive relationships and friendships within the community, which affords people happiness and self-worth. Groups within a community, such as Men’s Groups, Women’s Groups and sporting clubs, were all identified as offering valuable opportunities for developing friendships and receiving support and relief from stress.

*‘[Men’s group], it’s a place where we come, we’re usually laughing… having a good time… so, we all come to have a bit of a yarn and a bit of a laughter. It’s so good for you, mental health.’*

(Participant, New South Wales, Inner Regional)

*‘That’s why we get together as a group of women and we sit around and have a little yarn up and that and everything comes out and we talk about everything. You’re leaving your stress at home and you’re sitting there with your girlfriends and you’re talking and that’s a good thing about it. It’s just relaxing. …it’s support really, support… sister support… just wind down.’*

(Participant, New South Wales, Inner Regional)

Moreover, it was outlined as important that communities should have access to high quality and suitable services that support the wellbeing of all community members.

Participants identified that holistic health is promoted through engagement with a variety of cultural activities, such as, fishing, hunting, collecting bush tucker, traditional medicine and healing, singing, dancing, and songlines. Participating in cultural activities was also regarded as important to promoting physical health, as well as cultural and spiritual health and healing. Manifold benefits were identified from activities such as catching and sharing traditional foods. Participants found they were physically healthier due to the inherent physical activity associated with it and the nutritious quality of the food, as well as the mental and spiritual benefits from engaging in traditional culture and connecting with other people.

*‘They go out and get kangaroo meat and bring it back and share it, and share it to family, share it to community, the needy… so, yes, it is good.’*

(Participant, South Australia, Outer Regional)

*‘Like, looking back from when my dad did gardening, we eat food from the ground, not from the shops. He goes fishing, we eat fish. The only thing we buy from the shop would be a bag of rice and a tin of flour—bag of flour. And, everything else comes out of the ground… we grew up healthy.’*

(Participant, Queensland, Outer Regional)

Having a strong connection to traditional culture is said to give people self-worth, which makes people strong and is good for wellbeing. Knowing and talking about culture and identity with other people was reported as good for wellbeing.

*‘Having that culture gives you that self-pride or self-respect as well, which actually benefits you, well, your health, your mental health… no, it’s just like out there now, being a healthy lifestyle, like now they don’t even have smoke.’*

(Participant, Victoria, Major City)

Being prevented from embracing and sharing Aboriginal and Torres Strait Islander culture caused many problems with health and wellbeing.

*‘Like, I grew up, say, during the assimilation where you weren’t allowed to talk to anybody about anything; we kept our business to ourselves. And I see a big difference when I look at my nephews and nieces who talk about everything with family and I’m thinking—I think they’re better for it and I know what it’s like to be discriminated against and even though it happens–I know it’s still happening today. But I think, in a lot of cases, things are getting better, and I think that’s all important to your social work, emotional wellbeing.’*

(Participant, Queensland, Major City)

Passing on cultural knowledge about health and wellbeing to children was also identified as important for ensuring the wellbeing of future generations.

*‘And those boys, they do a lot of workshops with all the young ones… mentoring for the young ones. Dancing.’*

(Participant, New South Wales, Inner Regional)

#### 3.2.3. Purpose and Control

Participants identified that having a sense of purpose and a sense of control over one’s life is fundamentally important to their wellbeing. Purpose and control were commonly intertwined in the reports from participants. Most often, reflections relating to purpose and control were discussed by participants in the context of stability at home, employment and financial security, education, and cultural and familial responsibilities.

Family was spoken about as a key aspect in determining one’s sense of stability. A stable family life was considered important for individuals to feel safe and feel that they were in control of their life.

*‘So, having that stability in a job and having that reassurance that you’re able to give your family the best things or the things that they need is very important to one’s health.’*

(Participant, Queensland, Major City)

Furthermore, participants said that having a stable family life offers opportunities for adults to care for others, including children. Likewise, having a job and job security was said to enable individuals to look after their family and to be able to provide a healthy lifestyle.

*‘I think we need to have education so that we can, sort of, all get on the same page and once you’re settled in place, it follows on, and that leads you to employment and leads you to a healthy lifestyle because you can afford that and support that. If you haven’t got education, you’re left behind the eight-ball, playing catch up.’*

(Participant, Northern Territory, Outer Regional)

However, having a job was sometimes said to make it difficult for individuals to fulfil their cultural, community and family responsibilities, which can cause stress and reduce wellbeing. However, it was considered important for adults to show their children that hard work and education were also valued, so that the children could envisage a future for themselves.

*‘Lead by respect and that. It sets an example for them—to the young ones in the family growing up. Dad, uncle, going off to work every day. See, role model.’*

(Participant, Queensland, Remote)

Participants also identified that passing on cultural and spiritual knowledge to children is an important responsibility, the fulfilment of which can confer purpose and control to others, as well as themselves. It was explained that Aboriginal and Torres Strait Islander adults are regarded as the primary educators of children and the responsibility rests with them to ensure that cultural knowledge is passed on and does not die out. This was seen as an important purpose for Aboriginal and Torres Strait Islander adults.

*‘It’s very important to encourage our young kids to get a good education.’*

(Participant, Northern Territory, Outer Regional)

*‘I guess that making sure that my children go to school, they are in good health, I am working, and I find that whilst working and you are in a routine and everyone is healthy so it sort of just pans out.’*

(Participant, Northern Territory, Outer Regional)

The ability of Aboriginal and Torres Strait Islander people to fulfil these cultural and familial responsibilities was considered important to fostering wellbeing, both for themselves and for others.

*‘Having a stable environment around family and the kids is—it all boils back to—coming back to education. What we learn we pass on to our kids as much as possible and then at the end of the day they have the right to make choices.’*

(Participant, New South Wales, Outer Regional)

Being part of a community group was also put forward as an important aspect of participating in society and fostering a sense of purpose and intent. Community groups were said to offer individuals opportunities to have a voice, contribute their ideas, and take part in decision making in their community, all of which are beneficial to one’s sense of wellbeing.

*‘It’s a good network to be around as well… everyone’s got a voice and everyone’s listening. Being a part of a group… it’s pretty good. All the boys come along.’*

(Participant, New South Wales, Inner Regional)

Community groups were seen to facilitate participation in cultural education and experiences (e.g., hunting and sharing). These experiences can instil pride through developing particular skills or interests, and through building both purpose and control for those who participate.

*‘I think that having that cultural background, knowing who I am, where I’ve come from may help me where—to know where I’m going. And it’ll help me, push me to do good things, better things in order to help other people I suppose.’*

(Participant, Queensland, Major City)

Participants explained that they have a responsibility to pass on cultural knowledge and Lore (i.e., the traditional customs and stories that Aboriginal peoples learned from the Dreamtime) to future generations—including language, hunting, storytelling and maintaining traditional sites. Holding these cultural responsibilities was described as an honour and a privilege, and it was said that learning cultural knowledge is not simply given, it must be earned.

*‘Them young fellas, they don’t learn. You gotta take them out into the bush… show them the old ways, like we’d—how we grew up and what we learnt.’*

(Participant, Queensland, Outer Regional)

*‘So, I’ve got a responsibility to maintain that Lore and also to hand it on to others, you know? So, that’s a cultural responsibility, but it’s an honour as well and a privilege… responsibility of maintaining that Lore.’*

(Participant, New South Wales, Inner Regional)

Participants reported that their sense of purpose can be heightened when they are employed to work in a position where they are able to use and share cultural knowledge, and where this knowledge is highly valued. Such circumstances were regarded by participants as particularly beneficial for strengthening wellbeing.

While holding multiple roles and responsibilities in a community was said to strengthen one’s sense of purpose, competing priorities from work were identified as making it difficult to find a good balance. This was said to reduce the sense of control and cause stress, negatively impacting on wellbeing. Similarly, mainstream education was seen as necessary for people to have access to work opportunities, but this can come at the expense of time spent with family, and learning culture.

*‘So things are better for us, or for some of us, which is great, but from that cultural perspective it’s come at a price, …now you do need to have an education and to get into jobs where you can make decisions that are going to help community or affect community, you have to have a university degree.’*

(Participant, Tasmania, Inner Regional)

#### 3.2.4. Dignity and Respect

The importance of an individual feeling that they are perceived and treated by others with dignity and respect was foundational to wellbeing and was connected with: people’s relationships with others, policies, services, and experiences of racism.

The strength and pride that family connections can afford individuals was put forward as the bedrock of one’s dignity and respect. In the face of persistent injustice and immense challenges, people’s connections with family provided a source of shared strength that empowers and motivates.

Participants spoke of the desire for government policies to be made with consideration of the collective wellbeing, to ensure that Aboriginal and Torres Strait Islander people are treated respectfully and supported to thrive, via culturally appropriate policies and services.

*‘In terms of the policies that are made about us without us, sort of thing, is really important and somehow that needs to be addressed in terms of our quality of life… that’s really important to me as an individual, to my family and to our whole community.’*

(Participant, Tasmania, Very Remote)

Similarly, communities were said to offer an environment where people experience freedom, choice, empowerment, and mutual respect for one another. Participants explained that being around other Aboriginal and Torres Strait Islander people mutually strengthens their cultural connection and fosters a sense of pride and feeling valued.

Furthermore, being part of a community was said to provide opportunities to feel heard, to have a voice, and to share their views in a trusting environment. In addition, it was important to participants’ wellbeing that their communities should have a voice and be consulted on issues that they consider are important. Such consultation and inclusion in decision making was said to be central to the dignity of individuals and to the community as a whole.

*‘Because they’ve always got that statistics on the census, where they take all the information and they’ll have their big forums about to talk about our problems and why our life expectancy is—I mean, they don’t know… it’s more around not having that genuine engagement… they talk about us but they don’t hear us or something…’*

(Participant, New South Wales, Inner Regional)

*‘We’ve got no rights… it seems like it. We’re basically just shut out.’*

(Participant, New South Wales, Inner Regional)

Belonging to community groups was described as providing opportunities to connect with other people, to share life journeys and to enable advocacy for the entire community. Such opportunities were seen to build the strength of the community, and in turn build the strength and the dignity of its members.

*‘Community participation ties back into that sense of community. Empowerment, control, freedom, and choice, well, I think, in Australian society, that’s quite important and I realised the weight that’s put upon it, but I also realise that can be taken away from us at any point, just like that.’*

(Participant, Queensland, Major City)

The community was also said to provide culturally-appropriate services and help community members to access other required services. This was seen as affording people confidence and dignity in accessing services that they need.

Participants regarded enacting and maintaining their culture as a central component of achieving and promoting dignity and respect, which was explained as particularly significant for Aboriginal and Torres Strait Islander people, given their shared experiences of repression due to the ongoing effects of colonisation.

*‘Having that culture gives you that self-pride or self-respect as well, which actually benefits you, well, your health, your mental health…’*

(Participant, Victoria, Major City)

The loss of large amounts of traditional knowledge, languages and culture was outlined as evidence that Aboriginal and Torres Strait Islander cultures have not been valued in mainstream Australian society. The exclusion of Aboriginal and Torres Strait Islander languages, history and content in school curriculums was identified by participants as propelling this loss. These indications of an absence of respect and valuing of Aboriginal and Torres Strait Islander cultures was described as strongly affecting the wellbeing of Aboriginal and Torres Strait Islander people on many levels.

*‘I was never allowed to speak of [my Aboriginal culture] through my family so it was only bringing myself back into it and bringing my kids into the culture and stuff like that that they’re going to know, because no one spoke about it in our family.’*

(Participant, Tasmania, Inner Regional)

Greater inclusion of Aboriginal and Torres Strait Islander voices, stories, histories and languages in all areas of Australian society, including in schooling, was put forward by some participants as an important step to rebuilding the dignity of Aboriginal and Torres Strait Islander people.

*‘Now people are learning language and kids are learning language and just to be able to get up and do a welcome to Country in language… it’s so empowering and those things, I think, improve the health of the whole community because you can feel proud of those things.’*

(Participant, Tasmania, Inner Regional)

*‘We were taught at school that there were no Aborigines [sic] in Tasmania so… they need educating too… you can say, “Well, hang on, they are, they are there.” We’ve got an Aboriginal reserve just over the water there.’*

(Participant, Tasmania, Inner Regional)

Moreover, the presentation of Aboriginal and Torres Strait Islander people in media in more realistic and strength-focused ways, was regarded as critical to help reshape the perceptions of Aboriginal and Torres Strait Islander people.

*‘When you look in the media, there’s so much stuff—so much missed or limited information about oh, you know, this—these many Aboriginal people are on Centrelink (Centrelink is the Australian Government’s agency that delivers a range of Commonwealth services to the community, such as payments to the unemployed, carers, people with disabilities, and Indigenous Australians) and so, we look like—in the rest of Australia’s eyes, we look like the scum of society.’*

(Participant, Northern Territory, Outer Regional)

The cultural misappropriation of arts and cultural knowledge was described as an issue that damages wellbeing—both financially and via the disrespect this shows to the value of traditional cultural knowledge. Participants said that Native Title rights would give Aboriginal and Torres Strait Islander people better access to their traditional lands, which facilitates the practice of cultural activities that are essential to wellbeing.

*‘If we were able to gain proper Native Title over some of our land, that would enable us to carry out those cultural activities that our ancestors have been carrying out for maybe tens and more, thousands of years. Essential for our wellbeing, to my way of thinking.’*

(Participant, New South Wales, Inner Regional)

Experiences of racism, in all its forms, was described as greatly impacting the autonomy of the individual to practice cultural ways of life. The shame and disempowerment caused by racism inhibited the rights of Aboriginal and Torres Strait Islander people to live with dignity and respect.

*‘These stereotypes that were always on the TV about us… so, like it’s this big double standard that’s always out there and like I said, we are made to always feel like we are the problem. And what is underlying all those double standards? Racism… yeah… racism. Yeah and even with… racism, racism and racism.’*

(Participant, New South Wales, Inner Regional)

*‘If you wanted to get somewhere in life you had to be like a Caucasian person and act like that, speak like that, you know, couldn’t have your culture or anything… as a kid growing up, we were never allowed to speak the language…’*

(Participant, Northern Territory, Outer Regional)

The ongoing effects of racist actions and policies in Australia were regarded as having substantial negative impacts on the wellbeing of Aboriginal and Torres Strait Islander people generally. The pervasive and pernicious impact of racism on wellbeing for the participants was interwoven throughout many of the other themes. The daily grind associated with encountering and coping with overt and more subtle forms of racism served to diminish the full expression of hope and joy across many parts of life.

*‘…dealing with racism. That affects everything…’*

(Participant, Tasmania, Inner Regional)

Despite the immense pride that people felt about their culture, the sense of injustice and loss due to the slow rate of recognition and respect shown by Australian society was experienced as an ever-present affront, which greatly reduced wellbeing.

*‘…They painted our flag out here once, white, I came to work walking along, the Minister was coming over, Federal Minister [name] was coming over for a visit to open the park thing here… and I looked up and this flag, you couldn’t buy Aboriginal flags, I hand made that flag and they’d painted it white and it was the most destroying thing, most traumatic thing that I’ve suffered in my life, the impact of that… yeah, it was a process of trying to wipe us off the earth, get rid of the flag, get rid of them.’*

(Participant, Tasmania, Very Remote)

#### 3.2.5. Basic Needs

Participants expressed the imperative to have their needs, and the needs of their family and community met, in order to move forward and achieve good wellbeing. Basic needs identified by participants included: housing, money, access to services, education, employment, opportunities to thrive and justice.

The requirement for housing and accommodation that can meet the needs of large and fluctuating numbers of family and visitors was outlined as very important to an individual’s sense of wellbeing.

*‘They’re putting everyone in flats and people have got big families, they need homes… and family, you can’t really say no to family—homeless.’*

(Participant, Northern Territory, Remote)

Similarly, participants expressed the importance of being able to support their family members when needed—in terms of money and accommodation. It was also described as critically important that people should have the ability to attend cultural events, which requires sufficient money and transport.

*‘We need to look after our family who is the most important people in our lives, we need money. We need to provide it. That is why we are providers and protectors of our immediate family…’*

(Participant, Western Australia, Major City)

Many participants reported that they often struggled financially to meet their responsibilities, including: sending their children to the schools they would like, providing their families with good food, paying for things like rent, funerals and travel to see relatives and attend cultural events.

*‘I mean, we live in a low economic society, and for anything they want to compete with others, it’s a big ask. Yes, it’s a big ask for us to—having the kids to go to school even, to feed them with the low income, and the expectation of stretching that money to do other things as well.’*

(Participant, Queensland, Very Remote)

*‘What we get you know, to pay for the rent, what we are living in, if we have got a car to put the petrol in. So, if we have got to go to the doctor’s or we have got to go anywhere, we have got that. Enough to buy us to go and get a feed so we last from Monday to Monday.’*

(Participant, Western Australia, Major City)

The ability to be close to family was a key basic need for participants. Having access to services, including health, education and employment services, in the community meant that people could stay with their family and in the community. Also, participants reported that many families were broken apart when individuals were separated due to going to prison, or having children removed, and these were associated with the social circumstances and poverty facing some families. Participants also spoke of the need for communities to be safe and for supportive environments with access to services that supported their wellbeing. Without this, it was said to be impossible for people to thrive.

*‘Your environment that, not only the kids but ourselves are in, it impacts a lot on health, education, mental health, wellbeing, the whole lot. So, if you’re in a stable environment you’re quite capable of coping with mechanisms and sharing.’*

(Participant, New South Wales, Outer Regional)

## 4. Discussion

To our knowledge, this large qualitative study is the first nationally-relevant exploration of the foundations of wellbeing for Aboriginal and Torres Strait Islander adults. Through the participation of 359 Aboriginal and Torres Strait Islander adults residing across Australia, and an Indigenist research approach to the study design and analysis, five foundations of wellbeing were identified: belonging and connection; holistic health; purpose and control; dignity and respect; and basic needs. These foundations are interwoven by three interconnected aspects of Aboriginal and Torres Strait Islander life: family, community and culture.

Consistent with previous research [21,41,42,43,44,45,46], the centrality of family, community and culture to the wellbeing of Aboriginal and Torres Strait Islander people has been highlighted by our study. Our earlier work [9] and other previous findings [22,46,47] demonstrating the importance of the interconnectedness of wellbeing dimensions for Aboriginal and Torres Strait Islander adults have been further reinforced by this current work. These synergies with previous findings strengthen the notion that any consideration of wellbeing for Aboriginal and Torres Strait Islander people must account for the interconnectedness and importance of the family and community, which locates wellbeing beyond the confines of the individual.

Our research has identified components of wellbeing which have relevance to Aboriginal and Torres Strait Islander adults across the country. While an emerging evidence base around the wellbeing of Aboriginal and Torres Strait Islander adults exists, to date, research has been largely conducted in specific geographic locations, or with particular communities or language groups [19,20,21]. Our study extends this evidence to encompass a national perspective. Direct comparison to these more specific settings is somewhat hampered by the limited existing evidence; however, there are some notable parallels.

Belonging and connection emerged strongly in our study as a component of wellbeing. This is consistent with a growing research evidence base on the centrality of family and community to the wellbeing of Aboriginal and Torres Strait Islander people, most commonly focusing on the emotional benefits of relationships [20,21,48,49]. Our findings extend this evidence to include the role of these critical relationships in supporting identity and cultural survival. The importance of autonomy, control and representation to the wellbeing of Aboriginal and Torres Strait Islander individuals [43,50,51,52,53] aligns with aspects in two of the components (purpose and control, and dignity and respect). The prerequisite of having one’s basic needs fulfilled in order to achieve good wellbeing is commonly reported in the literature for this population, particularly in large-scale, quantitative analyses [54]. Issues around food security [49,55], money [56,57,58], and housing [49,59] have previously been flagged as important issues for the wellbeing of this population. Our findings suggest that the importance of these factors relates to their impact on an individual’s ability to support and connect with others. This finding has been identified in a few previous studies [59,60].

### 4.1. Strengths and Limitations

The Indigenist research approach used in this study has enabled the development of a robust and unique nationally relevant evidence base about the key components of the wellbeing of Aboriginal and Torres Strait Islander adults. Additionally, this study was greatly strengthened by the respectful collaboration of Aboriginal and Torres Strait Islander and non-Indigenous researchers, and was guided throughout by an Aboriginal and Torres Strait Islander Advisory Group.

The large sample of Aboriginal and Torres Strait Islander adults from across the country has contributed a large collection of views and experiences to the study data. We aimed to achieve a diverse sample from across the country; however, there were more participants from regional and remote areas than from major cities, and around half our sample was over the age of 50. The consistency of results across geographical areas and age groups however supports the underlying commonality in the foundations and experiences of wellbeing.

### 4.2. Implications

The views of this large and diverse sample offer a broad and inclusive perspective on the parts of life that are most valued by Aboriginal and Torres Strait Islander adults and form the foundations of their wellbeing. These findings provide a valuable evidence base about wellbeing that can inform a wide range of topic areas and applications, including: the development of quality of life and wellbeing measures, policy and practice in decision making, service provision and planning, and a variety of research endeavours. Our study makes an important and timely contribution to understanding what is important to the wellbeing of Aboriginal and Torres Strait Islander people. The iterative, Indigenist research methods employed in this study offer a robust and culturally-appropriate design for research conducted with Aboriginal and Torres Strait Islander people.

## 5. Conclusions

The WM2Adults Project has privileged the voices of Aboriginal and Torres Strait Islander people to gain an understanding of the foundations of wellbeing from their perspective. In contrast to existing policies, which are based on Western biomedically-grounded notions of health; policies and practices that are informed by our findings have the potential to address outcomes that are meaningful for Aboriginal and Torres Strait Islander people.

## Figures and Tables

**Table 1:** Socio-demographic characteristics of What Matters sample (*n* = 359).

	*n*	%
Age group		
18–19 years	12	3.3
20–29 years	48	13.4
30–39 years	50	13.9
40–49 years	64	17.8
50–59 years	69	19.2
60–69 years	71	19.8
70–79 years	28	7.8
80+ years	8	2.2
Missing	9	2.5
Sex		
Male	152	42.3
Female	205	57.1
Other	2	0.6
Missing	-	-
Education level		
Year 10 or below	193	53.9
Year 11 or 12	52	14.0
TAFE certificate/diploma, trade certificate	73	20.3
University	31	8.6
Missing	10	2.8
Aboriginal and Torres Strait Islander group		
Aboriginal	303	84.4
Torres Strait Islander	21	5.8
Both Aboriginal and Torres Strait Islander	23	6.4
Missing	12	3.3
Marital status		
Single	136	37.9
Defacto/married	153	42.6
Widowed	37	10.3
Separated or divorced	21	5.8
Missing	12	3.3
Main employment status		
Employed	126	35.1
Student	8	2.2
Retired/Pension	101	28.1
Home duties	24	6.7
Other	4	1.1
Not working	87	24.2
Missing	9	2.5
Main language at home		
English	289	80.5
Other language	39	10.9
Two or more languages	22	6.1
Missing	9	2.5
Annual household income		
Less than $25,000	176	49.0
$25,000–$40,000	63	17.5
$40,000–$80,000	68	18.9
More than $80,000	29	8.1
Don’t know	2	0.6
Missing	21	5.8
Remoteness		
Major city	57	15.9
Inner and Outer Regional	205	57.1
Remote and Very Remote	97	27.0

## Data Availability

Data may be available from authors on request.
